# Surgical site infection in severe trauma patients in intensive care: epidemiology and risk factors

**DOI:** 10.1186/s13613-024-01370-7

**Published:** 2024-09-02

**Authors:** Lucie Savio, Pierre Simeone, Sophie Baron, François Antonini, Nicolas Bruder, Salah Boussen, Laurent Zieleskiewicz, Benjamin Blondel, Solène Prost, Guillaume Baucher, Marie Lebaron, Thibault Florant, Mohamed Boucekine, Marc Leone, Lionel Velly

**Affiliations:** 1 Département d’Anesthésie-Réanimation - Marseille, Université Aix-Marseille, CHU Timone, Marseille, France; 2 Département d’Anesthésie-Réanimation - Marseille, Université Aix-Marseille, CHU Nord, Marseille, France; 3 Institut des Neurosciences de la Timone, Université Aix-Marseille / CNRS, UMR7289 - Marseille, Marseille, France; 4 Datascientist Department, Service d’Informatique Médicale, Université Aix-Marseille, CHU Timone, Marseille, France; 5 Facultés de Médecine et de Pharmacie, Aix-Marseille Université, APHM, MEPHI, IHU Méditerranée Infection, Marseille, France; 6 Service de chirurgie orthopédique, traumatologique et vertébrale, Université Aix-Marseille, CHU Timone, Marseille, France; 7 Assistance Publique - Hôpitaux de Marseille, AP-HM, Hôpital Universitaire Nord, Neurochirurgie Adulte, Chemin Des Bourrely, Marseille, 13015 France; 8 Service de chirurgie orthopédique et de traumatologie, hôpital Nord, chemin des Bourrely, Marseille, 13015 France; 9 Centre D’Etudes Et de Recherches Sur Les Services de Santé Et Qualité, Faculté de Médecine, Aix-Marseille Université, Marseille, 13005 France; 10 Department of Public Health, University Hospital of Marseille, Marseille, France

**Keywords:** Surgical site infections, Trauma, Risk factors, Microbiology, Outcomes

## Abstract

**Background:**

Severe trauma is the leading cause of disability and mortality in the patients under 35 years of age. Surgical site infections (SSI) represent a significant complication in this patient population. However, they are often inadequately investigated, potentially impacting the quality of patient outcomes. The aim of this study was to investigate the epidemiology of SSI and risk factors in severe trauma patients.

**Methods:**

We conducted a multicenter retrospective cohort study screening the severe trauma patients (STP) admitted to two intensive care units of an academic institution in Marseille between years2018 and 2019. Those who underwent orthopedic or spinal surgery within 5 days after admission were included and classified into two groups according to the occurrence of SSI (defined by the Centers for Disease Control (CDC) international diagnostic criteria) or not. Our secondary goal was to evaluate STP survival at 48 months, risk factors for SSI and microbiological features of SSI.

**Results:**

Forty-seven (23%) out of 207 STP developed an SSI. Mortality at 48-months did not differ between SSI and non-SSI patients (12.7% vs. 10.0%; *p* = 0.59). The fractures of 22 (47%) severe trauma patients with SSI were classified as Cauchoix 3 grade and 18 (38%) SSI were associated with the need for external fixators. Thirty (64%) severe trauma patients with SSI had polymicrobial infection, including 34 (72%) due to Gram-positive cocci. Empirical antibiotic therapy was effective in 31 (66%) cases. Multivariate analysis revealed that risk factors such as low hemoglobin, arterial oxygenation levels, hyperlactatemia, high serum creatinine and glycemia, and Cauchoix 3 grade on the day of surgery were associated with SSI in severe trauma patients. The generated predictive model showed a good prognosis performance with an AUC of 0.80 [0.73–0.88] and a high NPV of 95.9 [88.6–98.5] %.

**Conclusions:**

Our study found a high rate of SSI in severe trauma patients, although SSI was not associated with 48-month mortality. Several modifiable risk factors for SSI may be effectively managed through enhanced perioperative monitoring and the implementation of a patient blood management strategy.

**Supplementary Information:**

The online version contains supplementary material available at 10.1186/s13613-024-01370-7.

## Background

Severe trauma is the leading cause of disability and mortality in people under 35 years with a large male predominance [1]. In Europe, it causes between 46 and 126 deaths per 100,000 inhabitants, with 5 million deaths per year worldwide [1]. Healthcare-associated infections are common in these patients reaching an incidence around 30% during the intensive care unit (ICU) stay [2].

Surgical site infection (SSI) is defined as an infection occurring after a surgical procedure and represents a public health issue as it is responsible for a significant morbidity and mortality [3]. It is the one of the most common causes of healthcare-associated infection [4]. In addition, there is a significant increase in cost associated with SSI, which have been shown to be tripled in orthopedic surgery [5]. The indirect costs in terms of loss of activity have been predicted at 1.85 billion dollars in 2030 [6]. Severe trauma by itself represents a risk factor for SSI [7]. A study suggested a rate of 37% of infectious complications in severe trauma patients, including 18% of SSI. Skin breakdown associated with trauma fracture increases the risk of SSI [8, 9], notably when external fixation is required [10]. However, to our knowledge, no recent studies assessed the prevalence, the long-term mortality and risk factors of SSI in patients with severe trauma.

The primary aim of this study was to evaluate the incidence of SSI in severe trauma patients. The secondary aims were to assess the impact of SSI on patient outcomes such as mortality and length of hospital stay, describe the microbiological profiles, and to identify risk factors associated with SSI.

## Methods

### Recruitment of patients

We conducted a retrospective, multicenter study in two hospitals (CHU Timone; CHU Hôpital Nord) of an academic institution (Assistance Publique Hôpitaux Universitaires de Marseille (APHM), Marseille, France, between January 01, 2018 and December 31, 2019). We chose to study this specific time period for two reasons: firstly, to avoid potential bias from the COVID-19 pandemic, which may have impacted the care of these patients and deviated from the usual gold standard care; secondly, because prior to 2018, biological data were not yet integrated into our digital recording system. We included patients admitted to ICU for severe trauma with at least one point on the Vittel criteria [11] (these criteria identify severe trauma patients needing immediate surgery based on vital signs, injury type and severity, and patient factors such as age and pregnancy) and requiring orthopedic or spinal surgery within the first five days after admission, according to the ICD10 coding system (Appendix 1). Inclusion was conducted by a professional from the Medical Informatics Department of the “*Assistance Publique des Hôpitaux de Marseille*”, independently of the study investigators, and then by individual screening of the Computerized Patient Files.

### Data collection

We collected data relating to the initial management of patients in ICU and operating theatre, and later during their stay in the surgical ward.

#### Biological variables

The biological variables collected were serum concentrations of creatinine, albumin, glucose, CRP, procalcitonin, hemoglobin, platelets, leukocytes and neutrophils, lymphocytes, fibrinogen, prothrombin, hematocrit, arterial oxygen pressure (PaO_2_), and arterial lactatemia. These variables were collected from the computer database shared by the laboratories of our institution on the day of surgery (D0), on the third day after surgery (D3), and on the seventh day after surgery (D7).

#### Non-biological variables

The variables related to the patients’ medical history or initial lesion(s) were age, sex, simplified acute physiology score (SAPS2) (Appendix 2), pre-existing diabetes, immunosuppression (defined as the presence of immunomodulatory treatments, an active hematological pathology or HIV), obesity, smoking or allergy to beta-lactams, and the presence of an orthopedic or spinal injury associated with the injury for which the patient was operated.

The variables related to resuscitative, anesthetic or later non-critical care management were the number of blood products transfused during the hospital stay (red blood cells (RBC), plasma (FFP), platelets (UP)), invasive mechanical ventilation before surgery, the duration of mechanical ventilation, the need for pre-hospital tracheal intubation, the need for an extracorporeal membrane oxygenation (ECMO) support, the administration of selective digestive decontamination, the duration of arterial or central venous catheterization, the volume of fluid expansion, defined as a volume greater than 1 L, on admission to the ICU and in the operating room, during the first day. We also collected the need for vasopressive support, the surgeon’s experience, defined as more than two years’ experience in the center and the development of other infection during ICU stay.

### Ethical approval

As this was a retrospective study based on data from medical records and coding registries, ethical approval was not required. A regulatory statement was provided to obtain the data. (N°HG5ZSS)

### Analysis of SSI characteristics

SSI was searched for in the computerized patient files shared by the APHM center according to the standardized and validated criteria of the Center for Disease Control [12] (Appendix 3). SSI was defined by bone or soft tissue infections documented by a positive bacteriological sample within 30 days. The patients were thus classified into two groups according to the occurrence of SSI or not. The characteristics of SSI were collected including the site, the type of material, the use of empirical and directed antibiotic therapy (as well as the duration of antibiotic exposure), the time of follow-up and the number of days of treatment. We also collected the number of total and in-hospital deaths to evaluate the survival of our patients with severe trauma according to the occurrence of SSI.

### Statistical analysis

The data were tested for normality of distribution (Shapiro-Wilk test) and are presented as means and standard deviations for continuous variables or medians and inter-quartile ranges for non-continuous variables according to their distribution. Categorical variables are presented as n (%). The patients developing SSI (SSI group) were compared with those not developing SSI (controls). Comparisons between groups according to their outcomes and developments Fisher’s exact test or Student’s t-test according to their distribution. Pearson’s square correlation (R2) was used to assess the correlation of the different variables. Missing data were not replaced. The analysis was performed in a blinded manner. Univariate and multivariate logistic regression analysis were used to investigate risk factors associated with SSI. Variables with a *p*-value < 0.2 according to a univariate analysis and those considered to be clinically relevant were included in the multivariate model. To reduce an excessive number of independent variables and an unstable estimate in the final model, seven multivariate prognostic models were tested. Receiver operating characteristic (ROC) curves of the probabilities of SSI allowed to evaluate classification performance. The area under the ROC curve (AUC) of each model was calculated. The ROC curves for the variables for which the area under the curve was > 0.7 were selected. Higher the AUC, the better the model correctly classifying SSI. The final model was selected based on the highest AUC. Sensitivity and specificity for the optimal cut-off probability based on the Youden’s index were presented. All analyses were performed using JMP version 13.

## Results

### Population characteristics and biological variables

**Fig. 1:**
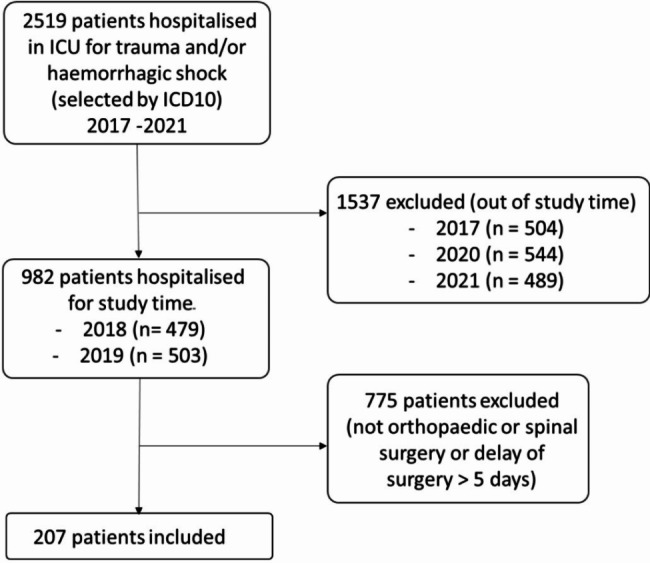
Flow-chart of the study

During the study period, 734 patients were admitted to the two ICUs for severe trauma (selected by ICD10) and 207 patients met the inclusion criteria. The flow-chart of the study is presented in Fig. 1.

Table 1 shows the patient features. Of the 207 patients included in our study, 47 (22.7%) developed an SSI. Within the first 5 days after ICU admission, 26% of spinal fractures and 82% of orthopedic fractures were treated in the operating room, with surgery in the first 24 h required for 71% of patients.

**Table 1:** Patient characteristics and features of their management

	Overall population (*n* = 207)	Controls (*n* = 160)	SSI patients (*n* = 47)	*p*
Median age	40 [26–57]	28 [26–57]	46 [30–55]	*0.36*
Male gender	167 (80.6%)	128 (80%)	39 (82.9%)	*0.41*
Median SAPSII score	**39 [30–49]**	**36 [30–47]**	**45 [39–52])**	***0.02***
Diabetes	14 (6.7%)	11 (6.8%)	3 (6.3%)	*0.66*
BMI > 30 kg/m^2^	8 (3.8%)	5 (3.1%)	3 (6.3%)	*0.26*
Smoking	54 (26.1%)	39 (23.3%)	15 (31.9%)	*0.20*
Immunosuppression	**9 (4.3%)**	**4 (2.5%)**	**5 (10.6%)**	***0.03***
Allergy to B-lactam	8 (3.8%)	7 (4.3%)	1 (2.1%)	*0.69*
Other infection during hospitalization	41 (19.8%)	32 (20%)	9 (19.1%)	*0.62*
Spinal fracture operated < 5 days	54 (26.1%)	43 (26.8%)	11 (23.4%)	*0.74*
Orthopedic fracture operated < 5 days	170 (82.1%)	128 (80%)	42 (89.3%)	*0.10*
Surgery < 24 h	159 (70.8%)	118 (73.7%)	41 (87.2%)	*0.08*
Selective digestive decontamination	55 (26.7%)	44 (27.7%)	11(23.4%)	*0.35*
Orotracheal intubation	71 (34.3%)	54 (33.7%)	14 (29.7%)	*0.44*
MV before surgery	101 (48.8%)	78 (48.7%)	23 (48.9%)	*0.56*
Mechanical ventilation > 24 h	69 (33.3%)	50 (31.2%)	19 (40.4%)	*0.16*
Arterial catheter > 24 h	175 (84.5%)	133 (83.1%)	42 (89.3%)	*0.21*
Central venous access > 24 h	169 (83.2%)	127 (80.9%)	42 (89.3%)	*0.07*
CRRT	3 (1.4%)	1 (0.1%)	2 (4.2%)	*0.13*
ECMO	5 (2.4%)	2 (1.25%)	3 (6.4%)	*0.08*
Epinephrine on arrival	116 (56.0%)	85 (53.1%)	31 (65.9%)	*0.08*
Epinephrine during surgery	125 (60.4%)	92 (57.5%)	33 (70.2%)	*0.08*
Epinephrine > 24 h	51 (24.7%)	35 (22.0%)	16 (30.0%)	*0.07*
Fluid expansion	**133 (64.25%)**	**96 (60%)**	**37 (78.7%)**	***0.02***
Number of RBC	**3 [1–6]**	**2 [0–5]**	**6 [3–10]**	***< 0.001***
Number of FFP	**0 [0–2]**	**0 [0–2]**	**2 [0–4]**	***< 0.001***
Number of PU	**0 [0–0]**	**0 [0–0]**	**0 [0–1]**	***0.004***
Length of stay (days)	17 [10–29]	17 [10–29]	17 [10–32]	*0.87*
Total cost (euros)	21 091 [17 088 − 27 686]	20 702 [17 093–27 400]	21 739 [16839–29765]	0.97
ICU cost (euros)	3 221 [1 610-8 040]	3 221 [1 610 -7 248]	3 221 [1 608 − 10 469]	0.97

Data are expressed as median [25th-75th quartile], mean (SD) or n (%); SAPSII = Simplified Acute Physiology Score II, BMI = Body Mass Index, MV = Mechanical Ventilation, CRRT = Continuous Renal Replacement Therapy, ECMO = Extracorporeal Membrane Oxygenation, RBC = Red Blood Cells, FFP = Fresh Frozen Plasma, PU = Platelets Units, ICU = Intensive Care Unit

### Comparison of patients with SSI and controls

Gender, age, history of diabetes, smoking, obesity, and delay < 24 h to undergo operating room were not associated with the SSI occurrence. The SAPS2 was higher in the SSI patients than in the controls (39 [14] vs. 46 [13]; *p* = 0.02)). A significant increase of SSI was reported in the immunocompromised patients, as compared with the non-immunocompromised patients (10.6% vs. 2.5%; *p* = 0.03). Of note, allergy to beta-lactams did not affect the occurrence of SSI (4.4% vs. 2.1%; *p* = 0.69) (Table 1).

The rate of other infections during hospitalization was similar in the two groups (20% vs. 19.1%; *p* = 0.62). Appendix 4 summarizes the different biological variables at D0, D3 and D7.

There was no statistically significant difference between the SSI patients and controls regarding the rate of pre-hospital tracheal intubation (34% vs. 30%; *p* = 0.44), the need for early invasive mechanical ventilation (48.7% vs. 48.9%; *p* = 0.56), and a duration of mechanical ventilation exceeding 24 h (40.4% vs. 31.2%; *p* = 0.16) (Table 1). The administration of selective digestive decontamination was not associated with a decrease of SSI (27.7% vs. 23.4%; *p* = 0.56).

The SSI group received a larger volume of fluid expansion, both at ICU admission (76.6% vs. 58.7%; *p* = 0.03) and in the operating room (78.7% vs. 60%; *p* = 0.02). They also received a higher number of RBC (6 [3–10] vs. 2 [0–5]; *p* < 0.001) and FFP (2[0–4] vs. 0[0–2]; *p* < 0.001). There was no difference in vasopressive support and surgeon experience.

### Analysis of microbiological profiles of SSI and antibiotic therapy

The distribution of SSI according to the different sites, materials, skin breakdown, delay of diagnosis, and microbiological documentation is presented in the Appendix 5 and 6. The preferential sites of SSI were tibia (27.6%) and femur (17%). Infection of the amputated limb accounted for almost 10.6% of SSI. The materials associated with SSI were external fixators and plates. Regarding the skin breakdown, 46.8% of SSI patients had a Cauchoix grade 3 fracture, 21.3% a Cauchoix grade 2 fracture and 25.5% a closed or Cauchoix grade 1 fracture. SSI occurred before 3 months, between 3 and 4 months and after 24 months in 83%, 15% and 2% of cases, respectively.

Regarding the microbiological documentation of SSI, 84.9% were performed using deep swabs and 25.5% using superficial swabs with the possible coexistence of these two types of swabs. Sixty-four per cent of SSI were polymicrobial with Gram-positive cocci and Gram-negative bacilli representing 72% of and 62% of pathogens, respectively. In 21% of SSI patients, the same pathogen was identified in another site.

Thirteen (28%) SSI patients received an empirical antibiotic treatment for less than 7 days, 25% for 7–14 days and 11% more than 14 days. Of note, data were missing in 36% of files. In 60% of cases, the directed antibiotic therapy was administered for a duration ranging from 1 to 3 months. A shorter duration was reported in 14% of cases while a prolonged course beyond 3 months was reported in 17% of cases.

Twenty-six per cent of pathogens had at least one acquired resistance. Of note, wild type and resistant bacteria were co-existing in 19% of patients with SSI. Empirical antibiotic therapy was active against the identified bacteria in 66% of cases (25.5% of data were missing on the choice of empirical antibiotic therapy) and ineffective in 8.5% of cases.

### Length of stay, mortality and economic analysis

**Fig. 2:**
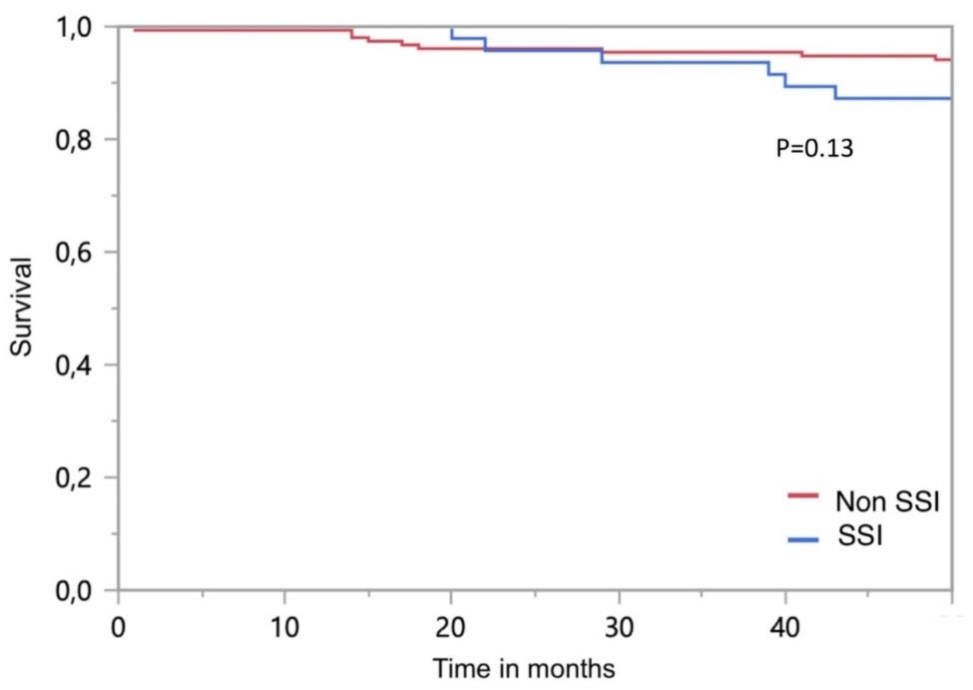
Kaplan-Meier analysis of survival in the two populations SSI and Non-SSI after 48 months

The length of hospital stay did not differ between with the patients with SSI and their controls (17 [10–32] vs. 17 [10–29] days; *p* = 0.87). The hospital and 48-month mortality rates did not differ between the two groups (Fig. 2). The total costs of the ICU and hospital stay were also similar in both groups (Table 2).

**Table 2:** Univariate analysis of risk factors for surgical site infection

	Odds-Ratio	CI for 95% Lower Upper		*P*-value
**Clinical features**				
Immune suppression	**3.199**	**1.019**	**10.037**	**0.046**
SAPSII	**1.032**	**1.008**	**1.056**	**0.009**
Fluid expansion	**2.467**	**1.146**	**5.31**	**0.021**
External fixation	**4.606**	**2.157**	**9.834**	**< 0.001**
Cauchoix 3 Grade	**7.808**	**3.568**	**17.085**	**< 0.001**
Use of RBC	**1.095**	**1.036**	**1.158**	**0.001**
Use of PU	1.05	0.774	1.425	0.755
Use of FFP	**1.058**	**0.993**	**1.127**	**0.08**
**Biological features at day of the surgery**				
Maximum lactate level	**1.114**	**1**	**1.242**	**0.049**
Maximum polynuclear level	1.032	0.983	1.083	0.199
Minimum hemoglobin level	**0.971**	**0.95**	**0.992**	**0.007**
Minimum platelets level	**0.992**	**0.986**	**0.999**	**0.021**
Minimum fibrinogen level	0.757	0.572	1.001	0.051
Minimum PT level	**0.964**	**0.945**	**0.985**	**0.001**
Minimum arterial oxygenation level_	**0.982**	**0.967**	**0.997**	**0.016**
Minimum albumin level	**0.913**	**0.859**	**0.971**	**0.004**
Maximum creatinine level	**1.014**	**1.005**	**1.023**	**0.002**
Maximum glycemia level	**1.082**	**1.002**	**1.169**	**0.043**
**Biological features at day 3**				
Minimum hemoglobin level	**0.944**	**0.911**	**0.977**	**0.001**
Minimum lymphocytes level	**0.375**	**0.157**	**0.896**	**0.027**
Minimum albumin level	**0.838**	**0.754**	**0.93**	**0.001**
Minimum PT level	**0.956**	**0.933**	**0.979**	**< 0.001**
**Biological features at day7**				
Minimum hemoglobin level	**0.947**	**0.916**	**0.979**	**0.002**
Maximum creatinine level	1.014	0.993	1.035	0.185
Minimum platelets level	0.998	0.995	1	0.106
Minimum arterial oxygenation level	0.983	0.959	1.007	0.17
Minimum albumin level	**0.898**	**0.814**	**0.991**	**0.033**

### Multivariate analysis

**Fig. 3:**
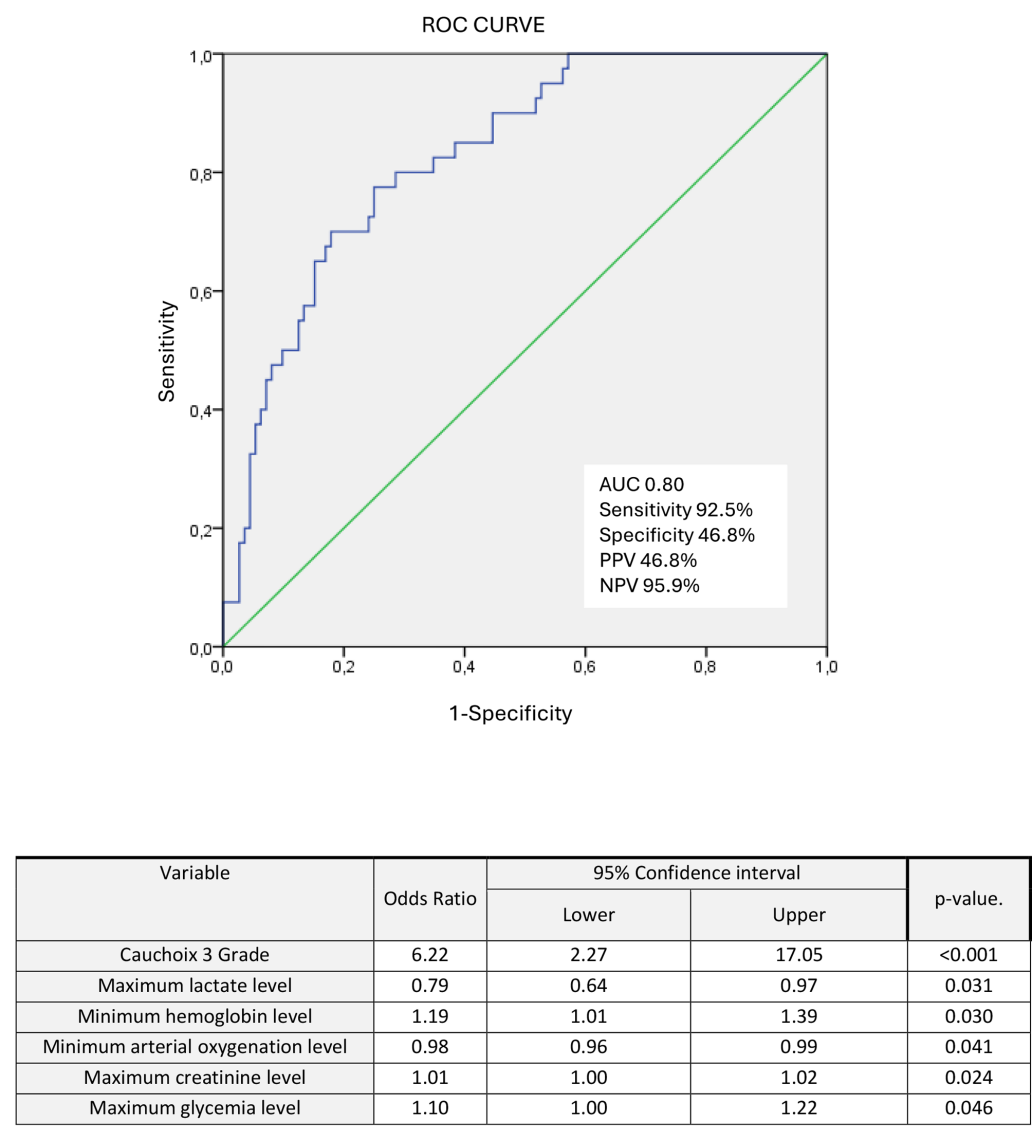
Characteristic of multivariate analysis of risk of developing SSI at the day of the surgery (model 3)

We included in the multivariate analysis the variables that significantly differed between the two groups in the univariate analysis (Table 2). On the day of surgery, low hemoglobin, low hematocrit, low arterial oxygenation levels, hyperlactatemia, high serum creatinine, high glycemia levels, and Cauchoix 3 grade fractures were associated with SSI (Fig. 3). The predictive model generated showed good prognosis performance with an AUC 0.82 [0.73–0.88], a sensitivity at 92.5 [80.1–97.4]%, a specificity at 62.5 [53.2–70.9]% and a NPV at 95.9 [88.6–98.5]%.

## Discussion

In our study, we observed an 23% incidence of SSI, which is higher than the rates previously reported in the literature. There was no difference in mortality at 48 months in both groups. Patients with SSI had polymicrobial infections primarily caused by Gram-positive cocci. Empirical antibiotic therapy was effective in 66% of cases. The independent risk factors for developing SSI were the presence of those factors on the day of surgery: low hemoglobin, low hematocrit and low arterial oxygenation levels, hyperlactatemia, high serum creatinine and high glycemia levels, and Cauchoix grade 3 fractures. The generated multivariate analysis predictive model demonstrated good prognostic performance.

In our study, we found almost 23% of SSI in severe trauma patients who underwent surgery within 5 days after ICU admission, which was higher than the standard SSI rates reported in recent years [13] since the literature showed rates of SSI in non-severe patients with open fractures up to 15% [14]. However, our population was at high risk to develop SSI [7, 15] and we highlighted several independent factors associated with an increase in the incidence of SSI that are particularly prevalent in ICU patients. This high incidence may be attributed to the severity of injuries and the complexity of the surgical procedures required.

Although we did not observe a difference in 48-month mortality in the two groups, the overall mortality rate was higher than typically reported in previous studies focusing on SSI, likely due to the severe nature of trauma in our ICU population. Severe trauma itself carries a significant risk of mortality, with studies reporting rates up to 45% in patients requiring ICU admission after major trauma [16]. Even in survivors after the initial injury, these ICU trauma patients remain at elevated risk of late mortality from complications like sepsis, organ failure, and nosocomial infections [2]. While SSI did not affect mortality, it may still interact with other important outcomes like functional status and quality of life, which were not evaluated here.

Certain management modalities, such as the administration of vascular filling, RBC and FFP transfusions, were more common in trauma patients with SSI [17]. This may reflect an increased severity of injuries, which was confirmed by higher SAPS II scores in the SSI patients. To our knowledge, the SAPS II has never been used to assess the risk of SSI in trauma patients. In contrast, the ISS score, a score measuring the severity of injuries dedicated to trauma patients, has not been shown to be correlated with the risk of SSI [7, 18, 19]. In addition, the surgeon experience and the use of selective digestive decontamination did not seem to influence the risk of SSI in agreement with other studies [20].

Microbiological analysis of SSI showed a high prevalence of polymicrobial infection. In the literature, this characteristic is often associated with skin breakdown [14], which was the case in our patients with a large number of open fractures. In our study, the majority of SSI occurred early in line with previous studies, in orthopedic surgery [6].

In addition, we found that the majority of infections were caused by Gram-positive cocci, which was already described in SSI [14]. We report several cases of SSI with antibiotic-resistant bacteria. In a French registry of SSI in orthopedic surgery, the proportion of methicillin-resistant *S. aureus* (MRSA) among *S. aureus* was 22% and the proportion of extended-spectrum β-lactamase-producing *Enterobacteriaceae* (ESBL) was 9%, with rates of ESBL increasing in recent years [21]. Our study found up to 45% of infections with at least one resistant germ, which seems important, but we included also acquired resistance in this category.

Low hemoglobin and hematocrit were independent risk factors for SSI. In a randomized controlled trial, Strobel et al. showed that preoperative anemia doubled the risk of SSI in digestive surgery [22]. In non-cardiac surgery, intraoperative transfusion was associated with an increase in mortality and postoperative complications including SSI [23]. it is difficult to know whether this adverse effect is due to the transfusion itself or to intraoperative blood spoilage and therefore anemia. This hypothesis has already been suggested in many surgeries [24]. In fact, previous studies defined anemia by a level of hemoglobin < 12 and 13 g/dl, excluding anemia < 9.5 g/dl [24], while our patients had minimum values that were mostly much lower. In a meta-analysis, Rohde et al. found an increase in healthcare-associated infections in almost 8,000 patients with a liberal transfusion strategy, supporting the hypothesis that transfusion increases the risk of infection independently of anemia [25]. Our univariate analysis suggested a link between the volume of transfusion and SSI. Severe bleeding and massive transfusions may have resulted in coagulopathy that could participate to the development of SSI [26].

Many studies have investigated the use of perioperative inspired oxygen fraction (FiO2) to reduce the risk of infection, with controversial results. One of the main hypotheses for this beneficial effect is that hyperoxia enhances the antibacterial action of polymorphonuclear neutrophils. However, hyperoxia may also lead to the formation of free radicals, with known cellular toxicity [27]. In 2016, the WHO recommended an FiO2 of 80% intraoperatively, with oxygen administration continued if possible, for 2–6 h postoperatively to reduce the risk of SSI [28].

Our study found an increased risk of SSI with higher glucose levels on the day of surgery. Hyperglycemia is common in severe trauma patients [29]. A meta-analysis of 15 randomized controlled trials suggested a reduction in SSI with strict glucose control (< 8 mmol/L) in cardiac, gastrointestinal, and intracranial surgery [30]. Guidelines recommended maintaining a perioperative glucose target of < 11 mmol/L to prevent SSI in all types of surgery [31]. In our study, the difference in glucose levels between the two groups was small but statistically significant.

Cauchoix grade 3 fractures are severe open fractures with extensive soft tissue damage and periosteal stripping. The large area of injury provides an entry point for bacterial contamination from the environment or patient’s skin flora. Devascularized tissues are susceptible to infection due to impaired immune cell migration and antibiotic penetration. Contamination is further exacerbated by the high-energy trauma mechanism and delayed presentation common in these injuries. Multiple studies have identified Gustilo-Anderson type III open fractures as a major risk factor for SSI after orthopedic trauma [7].

Our study has several strengths: few studies investigated the risk factors for SSI in severe trauma patients, although these infections are frequent. We included a relatively large number of patients in two ICUs from the same institution with a larger number of variables, making it possible to obtain a comprehensive overview of the SSI in this population. In addition, analysis included clinical, biological, perioperative, and economic studies with a focus on long-term mortality.

However, several limitations can be discussed. Firstly, variables such as the duration of surgery, intraoperative patient temperature or presence of associated injuries were not collected [32–34]. We did not find a significant increase of invasive mechanical ventilation in SSI patients, suggesting that severe brain injury was not associated with an increased risk for SSI. Secondly, we did not assess functional scores in our patients who can have an impaired quality of life due to SSI. Thirdly, we did not include SSI other than those involving the limbs or the spine, because we focused on bone surgery. Finally, the retrospective nature of this work may introduce methodological bias such as the unavailability of specific injury severity scores.

## Conclusions

Our study highlighted a high rate of SSI in severe trauma patients operated for bone fractures, with no effect on 48-month mortality rate. SSI were mainly polymicrobial with Gram-positive cocci, and empirical antibiotic therapy was effective despite the presence of resistant bacteria. The identified risk factors were low hemoglobin, hematocrit and arterial oxygenation levels, hyperlactatemia, high serum creatinine and glycemia levels on the day of surgery and severe open fractures. Several modifiable risk factors identified for SSI may be effectively managed through enhanced perioperative monitoring and the implementation of a patient blood management strategy. Further studies are needed to evaluate the long-term functional impact of SSI in this population.

### Electronic supplementary material

Below is the link to the electronic supplementary material.

Supplementary Material 1

## Data Availability

The datasets used and/or analyzed during the current study are available from the corresponding author on reasonable request.

## Abbreviations

SSI: Surgical Site Infections
ICU: Intensive Care Unit
STP: severe trauma patients
MRSA: Methicillin-Resistant Staphylococcus aureus
ESBL: Extended-Spectrum Beta-Lactamase
AKI: Acute Kidney Injury
