# Supplementary material for: Surgical site infection in severe trauma patients in intensive care: epidemiology and risk factors
Source: Ann Intensive Care. 2024 Sep 2;14:136. doi: 10.1186/s13613-024-01370-7 (PMC11366732; doi:10.1186/s13613-024-01370-7)
Supplement: Supplementary file 1 — Supplementary Material 1 [file 13613_2024_1370_MOESM1_ESM.docx]

SUPPLEMENTARY MATERIAL for

**Surgical site infection in severe trauma patients in intensive care: epidemiology and risk factors**

**Authors**: Lucie Savio, MD; Pierre Simeone, MD, PhD; François Antonini, MD; Sophie Baron, MD; Mohamed Boucekine, MD, PhD; Thibault Florant, MD; Nicolas Bruder, MD, PhD; Salah Boussen, MD, PhD; Laurent Zielesckiewicz, MD, PhD; Benjamin Blondel, MD, PhD; Solène Prost, MD; Guillaume Baucher, MD; Marie Lebaron, MD; Marc Leone, MD, PhD; Lionel Velly, MD, PhD

TABLE OF CONTENTS

[Appendix 1. National social security (CCAM) codes used for extraction of patient data and the corresponding ICD-10 codes (French Version) 3](#_Toc168066979)

[Appendix 2. Simplified Acute Physiology Score (SAPS II) 4](#_Toc168066980)

[Appendix 3. Diagnostic criteria for surgical site infections defined by the Centers for Disease Control, 1999 5](#_Toc168066981)

[Appendix 4. Biological variables at Day of surgery (DS), Third day of surgery (D3), and Seventh day of surgery (D7) 7](#_Toc168066982)

[Appendix 5. Probabilistic/appropriate antibiotic therapies and germs responsible for SSI 8](#_Toc168066983)

[Appendix 6. Detailed SSI characteristics 13](#_Toc168066984)

# **Appendix 1**. National social security (CCAM) codes used for extraction of patient data and the corresponding ICD-10 codes (French Version)

| S321 | Fracture of the pelvis |
| --- | --- |
| S5231,S4231 | Open fracture of the humerus, radius and/or ulna |
| S143 | Traumatic injury to the brachial plexus |
| S443 | Traumatic injury to a nerve in the upper limb |
| S450 | Traumatic arterial or venous injury to the upper limb |
| S579 | Crushing of the shoulder, arm, elbow or forearm |
| S47 | Crushing of other and unspecified parts of the shoulder and arm |
| S489 | Traumatic amputation of shoulder, arm, forearm, hand |
| S789 | Traumatic amputation of hip, thigh or knee |
| S889 | Traumatic amputation of leg, ankle or foot |
| T111 | Open wound of the upper limb |
| T131 | Open wound of the lower limb |
| S7291 | Open fracture of the femur, unspecified part |
| S7290 | Closed femur fracture, unspecified part |
| S8221 | Open fracture of tibia and/or fibula, unspecified part |
| S740 | Traumatic injury to a nerve in the lower limb |
| S850 | Traumatic arterial or venous injury to the lower limb |
| S3810 | Crushing of the buttock |
| S772 | Crushing of the hip and/or thigh |
| S870 | Crushed knee and/or leg |
| S789 | Traumatic amputation of hip, thigh or knee |
| S429 | Fracture of the shoulder girdle, part of it |
| S433 | Dislocation of parts of the shoulder girdle |
| S131 | Luxation of a cervical vertebra |
| S1290 | Closed cervical vertebral fracture |
| S220 | Fracture of a dorsal vertebra |
| S320 | Fracture of a lumbar vertebra |

# **Appendix 2**. Simplified Acute Physiology Score (SAPS II)

**
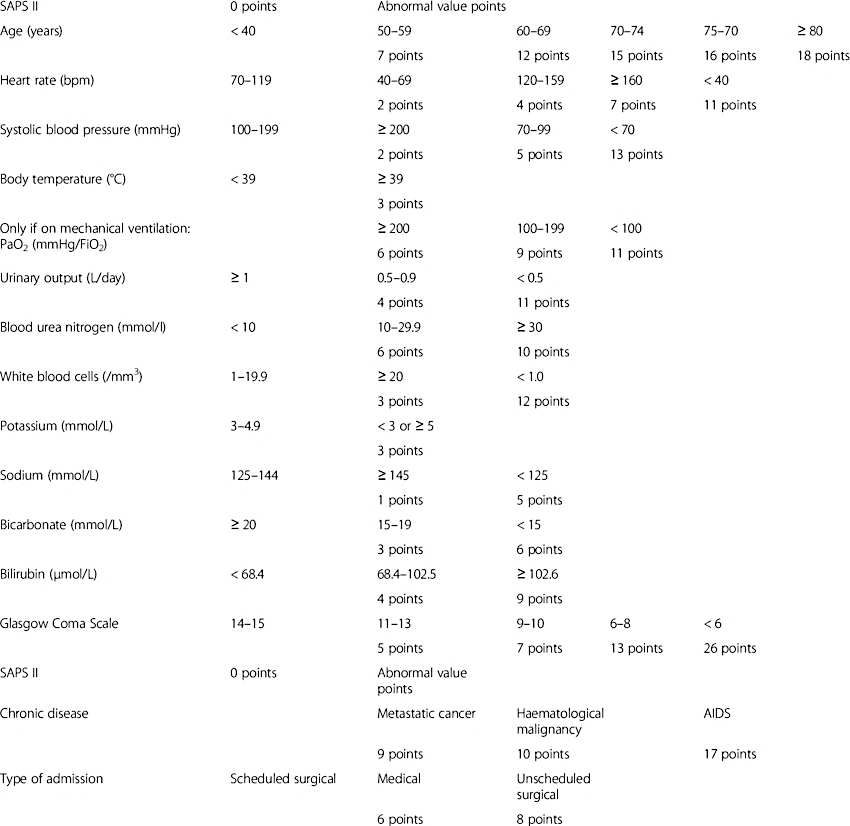
**

# **Appendix 3.** Diagnostic criteria for surgical site infections defined by the Centers for Disease Control, 1999

**SUPERFICIAL INCISIONAL SSI**

Infection occurs within 30 days after the operation and infection involves only skin or subcutaneous tissue of the incision and at leastone of the following:

1. Purulent drainage, with or without laboratory confirmation, from the superficial incision.

2. Organisms isolated from an aseptically obtained culture of fluid or tissue from the superficial incision.

3. At least one of the following signs or symptoms of infection: pain or tenderness, localized swelling, redness, or heat and superficial incision is deliberately opened by surgeon, unless incision is culture-negative.

4. Diagnosis of superficial incisional SSI by the surgeon or attending physician.

Do not report the following conditions as SSI:

1. Stitch abscess (minimal inflammation and discharge confined to the points of suture penetration).

2. Infection of an episiotomy or newborn circumcision site.

3. Infected burn wound.

4. Incisional SSI that extends into the fascial and muscle layers (see deep incisional SSI). Note: Specific criteria are used for identifying infected episiotomy and circumcision sites and burn wounds.

**DEEP INCISIONAL SSI**

Infection occurs within 30 days after the operation if no implant† is left in place or within 1 year if implant is in place and the infection appears to be related to the operation and infection involves deep soft tissues (e.g., fascial and muscle layers) of the incision and at least one of the following:

1. Purulent drainage from the deep incision but not from the organ/space component of the surgical site.

2. A deep incision spontaneously dehisces or is deliberately opened by a surgeon when the patient has at least one of the follow-

ing signs or symptoms: fever (>38ºC), localized pain, or tenderness, unless site is culture-negative.

3. An abscess or other evidence of infection involving the deep incision is found on direct examination, during reoperation, or by

histopathologic or radiologic examination.

4. Diagnosis of a deep incisional SSI by a surgeon or attending physician.

Notes:

1. Report infection that involves both superficial and deep incision sites as deep incisional SSI.

2. Report an organ/space SSI that drains through the incision as a deep incisional SSI.

**ORGAN/SPACE SSI**

Infection occurs within 30 days after the operation if no implant† is left in place or within 1 year if implant is in place and the infection appears to be related to the operation and infection involves any part of the anatomy (e.g., organs or spaces), other than the incision, which was opened or manipulated during an operation and at least one of the following:

1. Purulent drainage from a drain that is placed through a stab wound‡ into the organ/space.

2. Organisms isolated from an aseptically obtained culture of fluid or tissue in the organ/space.

3. An abscess or other evidence of infection involving the organ/space that is found on direct examination, during reoperation, or by histopathologic or radiologic examination.

4. Diagnosis of an organ/space SSI by a surgeon or attending physician.

*.*

# **Appendix 4.** Biological variables at Day of surgery (DS), Third day of surgery (D3), and Seventh day of surgery (D7)

|  | **Day of surgery (DS)** | | | **Third day of surgery (D3)** | | | **Seventh day of surgery (D7)** | | | |
| --- | --- | --- | --- | --- | --- | --- | --- | --- | --- | --- |
| **Biological variables** | **Non SSI (n= 160)** | **SSI (n= 47)** | ***p*** | **Non SSI (n= 160)** | **SSI (n= 47)** | ***p*** | **Non SSI  (n= 160)** | **SSI (n= 47)** | ***p*** |  |
| **Max blood glucose (mmol/L)** | **7,7 [6,9-9,4]** | **9,2 [8,1-12,3]** | ***<0,001*** | 6,3 [5,4-7,5] | 5,8 [5,1-7] | *0,14* | 5,9 [5,1-6,6] | 6 [5,2-6,7] | *0,60* |  |
| **Maximum creatinine (µmol/L)** | **81 [67-99]** | **100 [82-128]** | ***<0,001*** | 67 [57-78] | 72 [61-86] | *0,09* | **60 [51-70]** | **64 [56-74]** | ***0,01*** |  |
| **Min albumin (g/L)** | **29,5 [25,4-40]** | **27,2[23,1-28,5]** | ***0,003*** | **29 [25,3-32,3]** | **26 [23,3-27,9]** | ***<0,001*** | **30,4 [27,5-42,3]** | **26,7 [22-32,9]** | ***0,03*** |  |
| **PaO2 min (mmHg)** | **78 [68-92]** | **70 [63-80]** | ***0,01*** | 74 [65-86] | 72 [60-82] | *0,24* | 83 [67-98] | 77 [71-86] | *0,74* |  |
| **PaO2 max (mmHg)** | 188 [133-319] | 245 [157-347] | *0,10* | 97 [82-122] | 95 [82-114] | *0,79* | **109 [88-132]** | **87 [76-99]** | ***0,01*** |  |
| **Min hemoglobin (g/L)** | **87 [73-101]** | **80 [72-87]** | ***0,01*** | **81 [73-89]** | **74 [70-80]** | ***<0,001*** | **93 [85-101]** | **83 [75-96]** | ***<0,001*** |  |
| **Min hematocrit (L/L)** | **0,26 [0,21-0,3]** | **0,23 [0,21-0,26]** | ***0,004*** | **0,24 [0,21-0,26]** | **0,22 [0,2-0,24]** | ***<0,001*** | **0,28 [0,25-0,31]** | **0,25 [0,22-0,28]** | ***0,001*** |  |
| **Min PT (%)** | **71[61-81]** | **61 [45-73]** | ***<0,001*** | **85 [77-94]** | **73 [60-89]** | ***<0,001*** | 92 [85-98] | 86 [77-97] | *0,19* |  |
| **Platelets min (G/L)** | **139 [96-170]** | **113 [80-137]** | ***0,01*** | 131 [95-170] | 125 [84-155] | *0,19* | **357 [287-454]** | **312 [232-384]** | ***0,03*** |  |
| **Fibrinogen min (g/L)** | **2,61 [2,04-3,34]** | **2,16 [1,59-2,77]** | ***0,01*** | 5,84 [5,06-6,71] | 5,89 [4,95-7,13] | *1* | 6,72[5, 59 -7,99] | 7 [5,98-8,03] | *0,64* |  |
| **Leukocytes min (G/L)** | 9,45 [7,3-11] | 8,71 [7,5-11] | *0,35* | 8,4 [6,72-10] | 7,85 [6,57-10,22] | *0,28* | 12 [9,6-15] | 11 [9,25-13] | *0,43* |  |
| **Leukocytes max (G/L)** | **14 [10-18,14]** | **18 [12,5-20,7)]** | ***0,02*** | 9,62 [6,4-9,61] | 9,49[7,43-12,18] | *0,64* | 13 [9,7-16] | 11 [10-15,75] | *0,63* |  |
| **Neutrophils min (G/L)** | 7,7 [5,6-9,9] | 6,9 [5,67-9,04] | *0,24* | 6,44 [5-15-8,33] | 6 [5,31-7,2] | *0,41* | 8,7 [6,6-11] | 8,2 [7-10] | *0,76* |  |
| **Neutrophils max (G/L)** | **12 [8,5-16]** | **15 [9,5-17]** | ***0,03*** | 7,7 [5,7-10-14] | 7,14 [5,6-9,3] | *0,31* | 9,5 [6,9-12] | 8,6 [7,9-12] | *0,85* |  |
| **Lymphocytes min (G/L)** | 0,77 [0,54-1,08] | 0,73 [0,6-1,09] | *0,97* | **0,98 [0,66-1,3]** | **0,8 [0,53-1,09]** | ***0,03*** | 1,4 [1,04-1,9] | 1,3 [0,9-1-7] | *0,21* |  |
| **Max Lymphocytes (G/L)** | 1,4[1-1,9] | 1,5 [1,16-2,5] | *0,21* | 1,2 [0,9-1,5] | 1,1 [0,8-1,4] | *0,09* | 1,43 [1,1-1,9] | 1,4 [1,04-1,8] | *0,41* |  |
| **PCT max (ng/mL)** | 0,06 [0,03-0,3] | 0,055 [0,03-0,27] | *0,73* | 0,815 [0,6-11,9] | 3,4 [0,78-30,5] | *0,41* | 0,14 [0,09-0,35] | 0,45 [0,17-0,83] | *0,25* |  |
| **CRP max (g/L)** | 67,3 [6,2-163] | 57,8 [4,9-134,4] | *0,84* | 115,6 [67,7-233] | 183,4 [106-264] | *0,38* | 83,6 [39,5-130] | 104 [54,3-195] | *0,20* |  |
| **Max lactatemia (mmol/L)** | **2,2 [1,3-3,3]** | **3,4 [2,2-4,85]** | ***0,001*** | 0,95 [0,7-1,3] | 1,1 [0,7-1,5] | *0,47* | 1,1 [0,8-1,3] | 1,1 [1-1,84] | *0,27* |  |

*Data are expressed as median [25th-75th quartile], mean (SD) or n (%)*

# **Appendix 5**. Probabilistic/appropriate antibiotic therapies and germs responsible for SSI

| **SSI case** | **Probabilistic antibiotic therapy** | **Germ(s) responsible for ISO** | **Appropriate antibiotic therapy** |
| --- | --- | --- | --- |
| **1** | Imipenem/Cilastatin and vancomycin | Enterobacter *cloacae*  Staphylococcus *epidermidis* | Imipenem/Cilastatin and Vancomycin |
|  |  |  |  |
| **2** | Piperacillin/Tazobactam and Vancomycin | Staphylococcus *aureus* | Rifampicin and Ofloxacin  Clindamycin and Ofloxacin |
| **3** | - | Serratia *marcessens* | Piperacillin/Tazobactam Ciprofloxacin |
|  |  | Pseudomonas *aeruginosa* |  |
| **4** | - | S. *aureus* | Rifampicin and Fusidic acid |
| **5** | - | Escherichia *coli* | Amoxicilline and Clindamycin |
|  |  | Streptococcus *agalactiae* |  |
| **6** | Piperacillin/Tazobactam and Linezolid | Klebsiella *aerogenes* | Cefepime  Cotrimoxazole and Ciprofloxacin |
|  |  | Citrobacter *freundii* |  |
|  |  | P. *aeruginosa* |  |
| **7** | Piperacillin/Tazobactam and Vancomycin | Corynebacterium *amycolatum* | Amoxicillin and Clindamycin |
|  |  | Streptococcus *oralis* |  |
|  |  | S. *epidermidis* |  |
| **8** | Cefepime et Vancomycine | S. *epidermidis* | Rifampicin and Ofloxacin |
| **9** | Piperacillin/Tazobactam and Vancomycin | Streptococcus *anginosus* | Piperacillin/Tazobactam Ciprofloxacin and Clindamycin Amoxicillin and Clindamycin |
|  |  | S. *oralis* |  |
|  |  | P. *aeruginosa* |  |
|  |  | E. *cloacae* |  |
| **10** | Piperacillin/Tazobactam and Vancomycin | Klebsiella *pneumoniae* | Amoxicillin and Clindamycin Rifampicine |
|  |  | E. *coli* |  |
|  |  | Enterococcus *faecalis* |  |
| **11** | - | Entrobacter *spp* |  |
|  |  | E. *coli* |  |
| **12** | Piperacillin/Tazobactam and Vancomycin | S. *aureus* | Ofloxacine and Fusidic acid |
| **13** | Piperacillin/Tazobactam and Vancomycin | S. *aureus* | Teicoplanine et Clindamycine |
| **14** | Piperacillin/Tazobactam and Vancomycin | E. *coli* | Ceftriaxone et Ciprofloxacine |
| **15** | - | S. *aureus* | Cotrimoxazole et Ciprofloxacine |
|  |  | Micrococcus luteus |  |
|  |  | E. *coli* |  |
| **16** | Piperacillin/Tazobactam and Vancomycin | Aeromonas *eucrenophila* | Imipenem/Cilastatin et Ciprofloxacine et Clindamycine |
|  |  | Buttiauxella *gaviniae* |  |
|  |  | P. *aeruginosa* |  |
|  |  | E. *cloacae* |  |
|  |  | Aeromonas *veronii* |  |
|  |  | K. *aerogenes* |  |
| **17** | Piperacillin/Tazobactam and Vancomycin | S. *epidermidis* | Teicoplanin |
| **18** | Méropénem et Fluconazole | Haemophilus influenzae | Cefepime |
|  |  | Candida albicans |  |
|  |  | Lactobacillus rhamnosus |  |
|  |  | P. *aeruginosa* |  |
| **19** | Cefepime and Linezolid  Imipenem/Cilastatin and Vancomycin | E. *cloacae* | Cotrimoxazole and Ciprofloxacin |
|  |  | S. *epidermidis* |  |
|  |  | Enterococcus *hirae* |  |
| **20** | Imipenem/Cilastatin and Vancomycin | S. *aureus* | Imipenem/Cilastatin and Rifampicin and Ofloxacin  Minocyclin and Rifampicine and Ofloxacin |
|  |  | E. *cloacae* |  |
| **21** | - | E. *faecalis* | Amoxicilline/acide clavulanique et amoxicilline |
|  |  | K. *pneumoniae* |  |
| **22** | Piperacillin/Tazobactam | Citrobacter *koseri* | Cotrimoxazole and Ciprofloxacin |
|  |  | Enterobacter *cancerogenus* |  |
| **23** | Piperacillin/Tazobactam and Vancomycin | S. *aureus* | Clindamycin |
| **24** | - | E. *coli* | - |
| **25** | - | P. *aeruginosa* | Piperacillin/Tazobactam |
|  |  | C. *freundii* |  |
| **26** | Cefepime | Pseudomonas putida | Ceftazidime and Ciprofloxacin |
|  |  | E. *cloacae* |  |
| **27** | Piperacillin/Tazobactam and Vancomycin | S. *aureus* | Ceftazidime and Rifampicin andCiprofloxacin |
|  |  | P. *aeruginosa* |  |
|  |  | Finegoldia magna |  |
| **28** | Piperacillin/Tazobactam and Vancomycin | S. *aureus* | Teicoplanin and Clindamycin |
|  |  | Peptoniphilus harei |  |
|  |  | Peptoniphilus gorbachii |  |
|  |  | Aerococcus vaginalis |  |
|  |  | Proprionibacterium acnes |  |
| **29** | Piperacillin/Tazobactam and Vancomycin | S. *epidermidis* | Vancomycin and Ofloxacin et Rifampicin |
| **30** |  | S. *oralis* | Ceftriaxone and Rifampicin |
| **31** | Piperacillin/Tazobactam and Vancomycin | S. *epidermidis* | Rifampicin et Ofloxacin |
| **32** | Ceftriaxone | E. *coli* | Ceftriaxone and Ciprofloxacin |
|  |  | Finegoldia *magna* |  |
| **33** | Piperacillin/Tazobactam and Vancomycin | E. *coli* | Teicoplanin and Doxycyclin and Ceftriaxone  Doxycycline and Ceftriaxone |
|  |  | S. *epidermidis* |  |
| **34** | Piperacillin/Tazobactam and Vancomycin | S. *aureus* | Ofloxacin and Rifampicin |
| **35** | Piperacillin/Tazobactam and Vancomycin | P. *aeruginosa* | Ceftazidim and Ciprofloxacin and Teicoplanin |
|  |  | S. *epidermidis* |  |
| **36** | Piperacillin/Tazobactam and Vancomycin | E. *coli* | Ceftriaxone and Ciprofloxacin  Ciprofloxacin |
| **37** |  | S. *aureus* | Rifampicin and Ofloxacin |
|  |  | S. *epidermidis* |  |
| **38** | Cefotaxime and Gentamycin | - | Cefepime |
| **39** | Piperacillin/Tazobactam and Vancomycin | S. *aureus* | Ofloxacine and Rifampicin |
| **40** |  | S. *aureus* | Rifampicin and Amoxicillin and Levofloxacin |
|  |  | P. *acnes* |  |
| **41** | Piperacillin/Tazobactam and Vancomycin | S. *aureus* | Teicoplanin and Ceftriaxone  Ciprofloxacin and Doxycyclin |
|  |  | Staphylococcus *capitis* |  |
|  |  | E. *coli* |  |
|  |  | E. *cloacae* |  |
| **42** | Piperacillin/Tazobactam and Vancomycin | E. *coli* | Cotrimoxazole and Ciprofloxacin |
|  |  | S. *aureus* |  |
| **43** | Gentamycin et Clindamycin puis Piperacillin/Tazobactam | E. *cloacae* | Amoxicillin and Cotrimoxazole |
|  |  | E. *faecium* |  |
|  |  | C. *freundii* |  |
|  |  | E. *cancerogenus* |  |
| **44** | Vancomycin | C. *striatum* | Vancomycin |
|  |  | S. *epidermidis* |  |
| **45** | Piperacillin/Tazobactam and Vancomycin | S. *aureus* | Ertapenem and Ciprofloxacin  Ciprofloxacin |
|  |  | Finegoldia *magna* |  |
|  |  | E. *cloacae* |  |
|  |  | Enterobacter *bugandensis* |  |
| **46** | Amoxicillin/ Clavulanic acid | E. *cloacae* | Cotrimoxazole  Clindamycin and Doxycycline |
|  |  | S. *capitis* |  |
| **47** | Imipenem/Cilastatin and Vancomycin | E. *cloacae* | Imipenem/Cilastatin and Ciprofloxacin  Ciprofloxacin |
|  |  | S. *epidermidis* |  |

# **Appendix 6**. Detailed SSI characteristics

| **Site** | | **n (%)** | | |
| --- | --- | --- | --- | --- |
| Tibia | | | 13 (27,6%) |  |
| Femur | | | 8 (17%) |  |
| Foot/ankle | | | 6 (12,8%) |  |
| Stump | | | 5 (10,6%) |  |
| Pelvis | | | 4 (8,5%) |  |
| Spine | | | 4 (8,5%) |  |
| Hip | | | 3 (6,38%) |  |
| Elbow | | | 2 (4,3%) |  |
| Wrist | | | 1 (2,1%) |  |
| Patella | | | 1 (2,1%) |  |
| **Skin breakdown** | |  | | |
| Closed fracture or Cauchoix 1 | | | 12 (25,5%) |  |
| Cauchoix 2 | | | 10 (21,3%) |  |
| Cauchoix 3 | | | 22 (46,8%) |  |
| unspecified | | | 3 (6,4%) |  |
| **Material type** | |  | | |
| External fixator | | | 18 (38,3%) |  |
| Plate | | | 9 (19,2%) |  |
| Nail | | | 6 (12,8%) |  |
| Screws/stems | | | 6 (12,8%) |  |
| No material | | | 5 (10,6%) |  |
| Pins | | 3(6,4%) | | |
| **Onset time** | |  | | |
| Early (<3 months) | | 39 (83%) | |  |
| Delayed (3-24 months) | | 7 (15%) | |  |
| Late (>24 months) | | 1 (2%) | |  |
| **Type of samples** | |  | | |
| Deep | | 40 (84,9%) | |  |
| Superficial | | 12 (25,5%) | |  |
| **Type of germs** | |  | | |
| CGP | | 34 (72%) | |  |
| BGN | | 29 (62%) | |  |
| BGP | | 4 (8%) | |  |
| CGN | | 1 (2%) | |  |
| **Probabilistic antibiotic therapy duration** | |  | | |
| < 7d | | 13 (28%) | |  |
| 7-14d | | 12 (25%) | |  |
| > 14d | | 5 (5%) | |  |
| Unspecified | | 17 (36%) | |  |
| **Appropriate antibiotic therapy duration** | |  | | |
| < 1 months | | 7 (14%) | |  |
| 1-3 months | | 28 (60%) | |  |
| > 3 months | | 8 (17%) | |  |
| Unspecified | | 4 (9%) | |  |
| **Resistance** | |  | | |
| Sensitive (S) | | | 24 (51%) |  |
| Acquired resistance (R) | | | 12 (26%) |  |
| Coexisting SandR | | | 9 (19%) |  |
| Unspecified | | | 2 (4%) |  |
| **Probabilistic antibiotic therapy efficiency** | |  | | |
| Efficient | | | 31 (66%) |  |
| Inefficient | | | 4 (8,5%) |  |
| Unspecified | | | 12 (25,5%) |  |
